# Targeting pathogenic fibroblast-like synoviocyte subsets in rheumatoid arthritis

**DOI:** 10.1186/s13075-024-03343-4

**Published:** 2024-05-23

**Authors:** Hongyan Qian, Chaoqiong Deng, Shiju Chen, Xinwei Zhang, Yan He, Jingying Lan, Aodi Wang, Guixiu Shi, Yuan Liu

**Affiliations:** 1 Department of Rheumatology and Clinical Immunology, the First Affiliated Hospital of Xiamen University, School of Medicine, Xiamen University, 55th, Zhenhai Road, Xiamen, XM 361000 China; 2 Xiamen Municipal Clinical Research Center for Immune Diseases, Xiamen, XM 361000 China; 3 Xiamen Key Laboratory of Rheumatology and Clinical Immunology, Xiamen, XM 361000 China

**Keywords:** Fibroblast-like synoviocytes, Rheumatoid arthritis, Inflammation, Immune homeostasis, Therapeutic target

## Abstract

Fibroblast-like synoviocytes (FLSs) play a central role in RA pathogenesis and are the main cellular component in the inflamed synovium of patients with rheumatoid arthritis (RA). FLSs are emerging as promising new therapeutic targets in RA. However, fibroblasts perform many essential functions that are required for sustaining tissue homeostasis. Direct targeting of general fibroblast markers on FLSs is challenging because fibroblasts in other tissues might be altered and side effects such as reduced wound healing or fibrosis can occur. To date, no FLS-specific targeted therapies have been applied in the clinical management of RA. With the help of high-throughput technologies such as scRNA-seq in recent years, several specific pathogenic FLS subsets in RA have been identified. Understanding the characteristics of these pathogenic FLS clusters and the mechanisms that drive their differentiation can provide new insights into the development of novel FLS-targeting strategies for RA. Here, we discuss the pathogenic FLS subsets in RA that have been elucidated in recent years and potential strategies for targeting pathogenic FLSs.

## Introduction

Rheumatoid arthritis (RA) is one of the most common rheumatic diseases characterized by persistent synovial inflammation in multiple joints along with bone damage [1] and affects approximately 0.5 ~ 1% of the population worldwide [2]. Current drugs for treating RA including conventional synthetic disease-modifying anti-rheumatic drugs (csDMARD) and biological or targeted DMARDs (b/tsDMARDs) mainly target immune cells and inflammatory cytokines. Although b/tsDMARDs have greatly improved outcomes in patients with RA, approximately 40% of patients with RA do not respond to individual biologic therapies [3], and a significant proportion of patients with RA still have active disease and are considered as “difficult-to-treat RA” [4]. Additionally, general immune suppression by traditional immunosuppressive cell therapies can significantly increase the risk of infection, which remains a major challenge for RA management. A previous study showed molecular signature of synovium was different in patients who responded to IL-6 or CD20 respectively. Moreover, fibroblasts encoding gene signature was substantially increased in patients who showed no response to both IL-6 and CD20 targeted therapies [5]. Patients who do not respond to two biologics presented with a pauci-immune phenotype in the synovium [6]. These findings suggest that stromal cells are an emerging attractive new therapeutic target in RA.

Fibroblast-like synoviocytes (FLSs) are the main cell component in the inflamed synovium of patients with RA [7]. The synovium is composed of two layers: the intimal lining layer and the sublining layer [6]. In homeostatic states, the synovial lining is comprised of FLSs and macrophages (2–3 cell layers in total). In RA, the lining layer expands, and immune cells such as lymphocytes, macrophages and dendritic cells accumulate in the sublining layer [8]. Instead of sustaining homeostasis in the physiological state, FLSs in RA exhibit a “proinflammatory” or “aggressive/tissue damage” phenotype and increase the expression of inflammatory cytokines, chemokines and matrix metalloproteinases, leading to inflammation persistence and bone damage [8]. FLSs have been proven to play a central role in the pathogenesis of RA [9], and therapeutic strategies targeting FLSs might avoid systemic immunosuppressive consequences, in contrast with the immunosuppressive therapies. Thus, FLSs have long been considered as promising new therapeutic targets for RA [10]. Recent published reviews have summarized where do we stand in the era of FLS-targeted therapy [11, 12]. To date, no effective FLS-targeting therapies have been approved for use in the clinical treatment of RA, as fibroblasts are enriched in a wide array of tissues with multiple functions that are important for sustaining tissue homeostasis, and FLSs in the synovium cannot be easily distinguished from fibroblasts in other tissues by specific markers; thus, direct targeting of general fibroblasts markers in FLSs is a challenge for RA treatment.

With the help of high-throughput technologies such as scRNA-seq and CyTOF in recent years, our understanding of functionally distinct subsets of fibroblasts has been largely explored. Several pathological FLS phenotypes were found to be specifically expanded in RA. These novel RA-specific pathogenic FLS clusters might provide new promising therapeutic targets that could significantly decrease the side effects of general fibroblast-targeted therapies. Here, we review recent findings on pathogenic FLS subsets in RA and potential targeting strategies with the aim of providing a better understanding of the heterogeneity of FLSs in RA and new insights into FLS-targeted therapy.

## Pathogenic effects of FLSs in RA

In the physiological state, FLSs directly impact the synovial fluid composition by producing hyaluronic acid and other joint lubricants, such as lubricin (also known as proteoglycan 4). FLSs play an important role in helping shape and maintain the synovial extracellular matrix (ECM) by producing matrix components (such as fibronectin, type I and III collagens, vimentin, tenascin, proteoglycans and laminin) and ECM-degrading enzymes (such as proteases, matrix metalloproteinases, hyaluronan synthase, and cathepsins) [6, 13]. Additionally, FLSs under physiological conditions may have an anti-inflammatory/pro-resolving ability that helps sustain immune homeostasis in the local immune environment [14]. In RA, FLSs lose their homeostatic phenotype and acquire “*proinflammatory*” and “*aggressive/tissue-damaging*” phenotypes that mediate the persistence of inflammation and cartilage/bone damage. The pathological effects mediated by RA-FLSs include the following: (1) enhanced migration and proliferation with reduced apoptosis, which results in hyperplastic rheumatoid pannus formation and leads to direct cartilage and bone damage; (2) overproduction of matrix metalloproteinases (MMPs) (such as MMP1, MMP3 and MMP13), aggrecanases (ADAMTS4 and ADAMTS5) [9], and RANKL [15], which damage the collagen-rich structures of joint tissues and promote osteoclast differentiation; (3) overproduction of proinflammatory cytokines (such as type 1 interferons [16], IL6 [17]) and chemokines (such as CCL5, CCL8, CXCL5 and CXCL10 [18]) that direct the recruitment of immune cells into joints; (4) promotion of T-cell [19–21] and B-cell [22–24] activation and differentiation. The various physiological and pathogenic effects of FLSs suggest their functional heterogeneity.

## Pathogenic FLS subsets in RA

The FLS subsets in RA reported in recent studies are summarized in Table 1 [14, 25–34], and the phenotypic characteristics of the pathogenic FLS subsets are described. Among those markers, cadherin-11 (CDH-11), fibroblast activation protein α (FAPα) and podoplanin (PDPN/GP38) are considered as general markers that associated with the pathogenic FLS phenotype in RA [14, 35]; however, FLSs in the physiological state may also express these markers but at relatively low levels. CDH-11 was reported to be a relatively specific marker of FLSs compared with fibroblasts in other tissues [36, 37]. CDH-11 regulates the production of several proinflammatory cytokines, such as IL-6 [38]. CDH-11 knockout mice were resistant to joint inflammation and cartilage erosion, suggesting the vital role of CDH-11^+^ FLSs in the pathogenesis of RA [36]. PDPN is expressed predominantly on the lining layer, and studies have shown that a small number of FLSs in the sublinling layer also express PDPN [25, 29]. PDPN^+^ FLSs were found to be expanded in RA but not in OA or healthy synovial tissue [14, 39]. PDPN^+^ FLSs could migrate and invade cartilage in a mouse model of cartilage destruction, suggesting that PDPD^+^ FLSs are pathogenic and capable of invasion and destruction [25]. Previous research has demonstrated that PDPN^+^CD45^−^CD31^−^ cells, termed PRIME cells, can be found in peripheral blood and are similar to PDPN^+^ FLSs. The proportion of PRIME cells increased before RA flare-up, thus verifying the important role of PDPN^+^ FLSs in RA pathogenesis [40]. FAPα is considered as a marker of fibroblast activation. In RA, FAPα colocalized with PDPN in the synovium. Moreover, FAPα plays an important role in remodeling the immune environment by mediating the interaction of FLSs with immune cells, regulating cytokine secretion and initiating the immune response [41], and deletion of FAPα^+^ FLSs ameliorated both inflammation and bone erosion in a mouse model of arthritis [29].

**Table 1:** Functionally different FLS subsets in RA

**Reference**	**Subset**	**Location**	**Characteristics**	**Functional assay**
2016 [25]	PDPN^+^	Mainly in lining. **Increased in RA synovium.**	Capable of vascular transmigration from primary site to distant cartilage sites where they attach and invade cartilage. TNF-α or IL-1 stimulation in vitro increased PDPN expression.	Invade and degrade cartilage in the SCID mouse model of cartilage degradation. Migrate to secondary site at early time.
	CD248^+^	Restricted to sublining	TGFβ1 stimulation in vitro increased CD248 expression. Did not invade the implants of cartilage in vivo.	Migrate to secondary site at later time.
2018 [26]	CD90^-^CD34^-^	Lining	High expression of MMP1, MMP3, PRG4, HAS1 and CD55.	Coculture with peripheral blood monocytes led to increased number of TRAP-positive osteoclastic cells in vitro.
	CD34^+^	Lining and sublining Higher in swollen joints	Characterized by the expression of inflammatory cytokine genes IL6, CXCL12, and CCL2; increased expression of genes involved in fibroblast migratory response (CTHRC1, TWIST1, POSTN, LOXL2, PDGFRB and MMP14).	Exhibited enhanced in vitro invasion and migration in response to PDGFBB in vitro; recruit more peripheral blood monocytes in a transwell leukocyte recruitment assay.
	CD90^+^CD34^-^	Sublining (localize to perivascular zone) **Expanded in RA, higher in swollen joints.**	CDH-11^+^. Positively correlated with the proportion of infiltrated leukocytes in synovium. Enrichment of mitotic and proliferative genes; increased expression of genes involved in fibroblast migratory response (CTHRC1, TWIST1, POSTN, LOXL2, PDGFRB and MMP14); high expression of RANKL and low expression of TNFRSF11B;	Exhibited enhanced in vitro invasion and migration in response to PDGFBB in a transwell matrix invasion assay; coculture with peripheral blood monocytes led to increased osteoclastic cells.
2018 [27]	CD55^+^	Lining	Enrichment in expression of hyaluronan synthase 1 (HAS1); Enrichment in genes involved in pathways associated with endothelial cell proliferation and regulation of reactive oxygen species responses.	ND
	CD90^+^	Sublining	Enrichment in genes involved in pathways associated with MMP activity and organization of the extracellular matrix.	ND
2019 [28]	CD55^+^	Lining	High expression of lubricin (encoded by PRG4); High expression of DNASE1L3, a gene whose loss of function is associated with RA;	ND
	CD90^+^CD34^+^	Sublining	Express genes related to the extracellular matrix	ND
	CD90^+^HLA-DRA^high^	Sublining **Expanded > 15-fold in leukocyte rich RA compared with OA.**	CD34^-^. Express genes related to the extracellular matrix; Express genes related to MHC class II presentation and the IFNγ-mediated signaling pathway (such as IFI30); high expression of CXCL12, CXCL9; may contribute the main source of IL-6 production.	ND
	CD90^+^DKK3^+^	Sublining	Express genes related to the extracellular matrix; High expression of DKK3, CADM1 and COL8A2	ND
2019 [29]	PDPN^+^FAPα^+^CD90^-^	Lining	**Bone-effector profile;** High expression of CCL9 and TNFSF11, potent inducers of osteoclast activity; High expression of MMP3, MMP9 and MMP13; Surface expression of RANKL; Secrete high levels of RANKL; Have an increased RANKL: osteoprotegerin ratio; correlated with cartilage damage.	Stimulate osteoclast differentiation and activation in vitro; Mediate bone and cartilage damage and promote osteoclast activity when transferred into the joints of mice with STIR
	PDPN^+^FAPα^+^CD90^+^	Sublining **Expanded in RA**	**Immune-effector profile**; High expression of genes encoding cytokines and chemokines (including IL6, LIF, IL33 and IL34); correlated positively with severity of joint inflammation and bone erosion.	Transfer of these cells into mice with CIA increases CD4^+^ T cell, neutrophil and macrophage infiltration and reduces FOXP3^+^ regulatory T cells. Transfer of these cells into mice with STIR exacerbates disease.
2020 [30]	PRG4^+^	Lining	Constitute the majority of stromal cells in OA.	ND
	CD90^+^	Sublining (perivascular zone, close to endothelium) **Expanded in RA.**	Enriched in Notch activation signature. Expansion in RA was driven by endothelium derived Notch3 ligands.	Notch inhibitor DAPT blocked the differentiation of CD90^high^ FLS in vitro. Genetic deletion of Notch3 or the blockade of NOTCH3 signaling attenuates inflammation and prevents joint damage in mice with inflammatory arthritis.
2020 [31]	-	Lining	High expression of MMPs in active RA. Expression of MMPs was reduced and mediators of tissue repair and resolution (for example, IGFBP5/6, AXL) were increased in RA in remission.	ND
	HLA^high^	Sublining	ND	ND
	CD90^high^	Sublining	ND	ND
	CD90^+^CXCL14^+^	Sublining	Express GAS6, and this expression was increased in RA in remission. GAS6 derived from this subset may contribute to the homeostatic regulatory functions of lining-layer MerTK^+^ macrophages.	ND
	CD90^+^CD34^+^	Sublining	ND	ND
2022 [14]	Smoc2/Col15a1^+^	Sublining	**Homeostatic state**: CD90^+^. Marked by the expression of Smoc2, Thbs1, Vwa1, Col15a1 and genes that encode matricellular proteins and the BMP coreceptor Rgma, along with BMP/SMAD signaling pathways; expression of genes associated with steroid metabolism, including the cortisone-conversion enzyme Hsd11d1, suggesting an anti-inflammatory role. **During arthritis**: Positively regulate fibroblast migration and apoptotic processes. disappearance of characteristic steroid biosynthetic process, BMP signaling, and chondrogenesis.	ND
	Comp/Sfrp1^+^	directly adjacent to the lining,	**Homeostatic state**: CD90^+^. High expression of WNT modulators Dkk2 and Sfrp1, enrichment in WNT-mediated responses, TGF activity, and osteogenesis. the specific expression of Ecrg4 indicates a role in regulating tissue repair processes. **During arthritis**: exhibit phosphatidylinositol 3-kinase signaling; positive regulation of developmental growth, Wnt regulation, epithelial-to-mesenchymal transition; androgen receptor signaling are gradually reduced.	ND
	Osr1/Nr2f2^+^	adjacent to the lining	**Homeostatic state**: CD90^+^. Gene expression is linked to joint morphogenesis and reparative processes.	ND
	Meox1/Clu^+^	sublining	**Homeostatic state**: characterized by BMP signaling pathway activation and osteoblast and myoblast differentiation. Characteristic gene expression involves the Klf5, Clu, Id1, and Meox1 genes. **During arthritis**: exhibit phosphatidylinositol 3-kinase signaling. participate in osteoclast differentiation.	ND
	Dkk3/Lrrc15^+^	both sublining and lining **specific in arthritis**	**CD90**^**+**^**Prg4**^**high**^, most expanded in the hTNFtg joints. Express highly important genes for joint pathology including the ECM component Fbln7, Thbs4, Cthrc1, lrrc15, TF Runx1. Expressed genes suggesting multiple biological processes including regulation of immune and redox response, cell fate determination, and ECM remodeling, which indicate a multipotent transcriptional signature.	ND
	Dpp4/Pi16^+^	sublining	**Homeostatic state**: The gene expression signature indicates that these SFs drive processes relative to vasculogenesis and regulation of type 2 immune responses and myeloid lineage differentiation and homeostasis. Characterized by the expression of Pi16, Sema3c, Efemp1, and Dpp4. **During arthritis**: exhibit phosphatidylinositol 3-kinase signaling. engage in blood vessel remodeling. responses to hypoxia, the regulation of TGFβR signaling, the development of cartilage, and the type 2 immune responses are absent.	ND
	Prg4^high^/Tspan15^+^	lining	**Homeostatic state**: Prg4^high^CD90^-^, high express genes as markers of lining SFs such as Tspan15, Hbegf, and Htra4. **During arthritis**: expansion markers of inflammatory response (Ccl2, Ccl5, Hmox1 Saa3), class I antigen presentation (H2-K1, B2m, H2-Q7), and ECM remodeling (Mmp3, Timp1, Cd44)	ND
	Birc5/Aqp1^+^	both sublining and lining **specific in arthritis**	**CD90**^**+**^**Prg4**^**high**^, **increase in the hTNFtg joints.** express Mki67, PDGFα, Birc5, Aqp1, Acta2, the C1qtnf3 adipokine, and other chemokines such as Cxcl5, as well as several adhesion molecules. The functional annotation related to increased proliferating capacity, adhesion, and peptidase activity.	ND
	Pxt3/Notch3^+^	Sublining, around the vascular cells	**Homeostatic state**: activation of cytokines and chemokine pathways (Ccl7, Cxcl10, IL6, and Ptx3) and are associated with immune-regulatory functions including response to IFN-beta/gamma and LIF. Notch3 mainly in this cluster in normal state. **During arthritis**: exhibit PI3K signaling, participate in osteoclast differentiation, regulate monocyte differentiation and uniquely present activation of protein kinase B activity, positive regulation of stress-activated MAPK cascade, and positive regulation of response to hepatocyte growth factor, regulation of several cytokine responses and tissue regeneration were lost.	ND
2022 [32]	PARC^+^COL3A1^+^	Perivascular	**Vascular-interacting subsets**. Closely resemble the **CD90**^**+**^**NOTCH3-activated FLS**. Marker genes were enriched in pathways centered around ECM binding (includes COL11A1, SPARC, and LRRC15) and disassembly (includes MMP13, MMP11, and FAP) and developmental pathways (COL3A1, COL1A1, COL5A1, and TGFB1), may play a role in vascular remodeling. Notch signaling is specific to this subset.	ND
	CXCL10^+^CCL19^+^	Next to CD3^+^ T-cell-regions	**Immune-interacting subsets**. Enriched in genes for pathways involved in direct interaction with immune cells, including lymphocyte chemotaxis (CCL19, CCL2, and CCL13), antigen presentation (CD74, HLA-DRA, and HLA-DRB1), and positive regulation of T-cell proliferation (TNFSF13B, VCAM1, and CCL5). Show broad evidence of response to the pro-inflammatory cytokines interferon (IFN) g, IFN a, TNF-a, IL-1 and IL-12. Overlapped significantly with CD90^+^HLA-DRA^high^ FLS.	ND
2022 [34]	CD90^-^PRG4^+^	Lining **Highest in the myeloid pathotype**	Express high level of CD55, MMP3, and FN1. Enriched for pathways such as ‘focal adhesion’, ‘ECM-receptor interaction’ and ‘mineral absorption’; correlated positively with disease severity parameters in the fibroid pathotype.	ND
	CD90^low^ CXCL12^+^	Sublining **Most prominent in fibroid pathotype.**	Express high level of CXCL12, CCL2 and ADAMTS1, enriched for pathways associated with proinflammatory states (TNF signaling pathway, MAPK signaling pathway).	ND
	CD90^intermediate^ POSTN^+^	Sublining **Most prominent in fibroid pathotype.**	Express high level of collagen genes. Pathways unique for this subset are “human papillomavirus infection” (included genes: COL1A1, COL3A1, COL1A2, ACTN1, LAMB1) and “regulation of actin cytoskeleton” (included genes: ITGA10, MYLK, ACTN1, MYH10, PDGFRB, MYL9, ENAH, ITGA11, ITGB5). Positively related with tender and swollen joint counts and CRP levels in the lymphoid pathotype.	ND
	CD90^high^CXCL14^+^	Sublining	Express high level of CXCL14, C3, CD34, ASPN; enriched in pathways of “phagosome”, “gap-junction” and “arachidonic acid metabolism”; negatively associated with disease severity in all pathotypes.	ND
2024 [33]	CD200^+^ DKK3^+^	Sublining	Express high level of Cdh11, DKk3 but not Lrrc15	Conjunction with ILC2s and play a pivotal role in establishing a pro-resolving network.
	MMP3^+^/IL6^+^	sublining	Express high level of MMP3 or IL-6	Colocalized with pro-inflammatory immune cells in regions with an active inflammatory phenotype

**Fig. 1:**
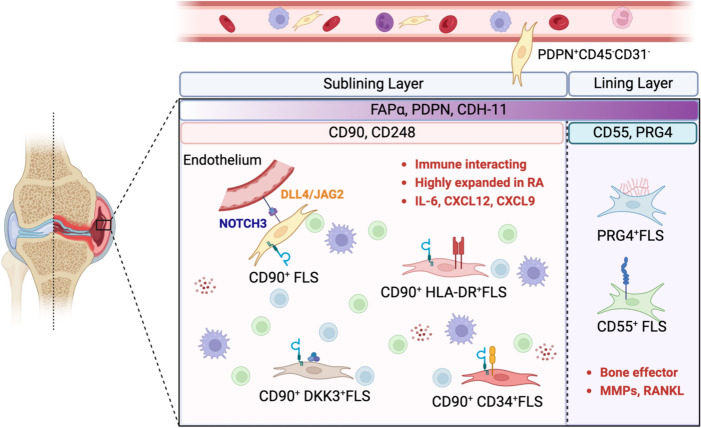
Pathogenic FLS subsets in RA. FLSs in the inflamed synovium can be anatomically distinguished into lining FLSs and sublining FLSs. FAPα, PDPN, and CDH-11 are expressed mainly on lining FLSs and also on sublining FLSs. The expression of FAPα, PDPN, and CDH-11 may resemble the pathogenic FLS phenotype, as targeting these markers can ameliorate arthritis in animal models of RA. Pathogenic FLSs in RA can be further subclassified into immune-interacting FLSs and bone-effector FLSs based on the expression of CD90 and CD55/PRG4, respectively. CD90 is expressed on sublining FLSs and can be used to designate proinflammatory FLS subsets that mediate inflammation persistence. Among CD90^+^ FLSs, a subset with high HLA-DRA expression that can secrete several proinflammatory cytokines and chemokines is substantially expanded in RA patients

Pathogenic PDPN^+^ or FAPα^+^ FLSs can be further divided into functionally distinct pathogenetic subsets. In 2019, Adam P Croft et al. [29] classified FAPα^+^PDPN^+^ FLSs into proinflammatory (CD90^+^FAPα^+^PDPN^+^, sublining) and bone damage (CD90^−^FAPα^+^PDPN^+^, lining) subsets based on the expression of CD90. Injection of CD90^+^FAPα^+^PDPN^+^ FLS into the inflamed ankle joint of mice led to more severe and persistent joint swelling, with greater leukocyte infiltration. In contrast, injection of CD90^−^FAP^+^PDPN^+^ FLSs led to increased osteoclast activity and joint damage but did not affect the severity of joint inflammation. Similarly, several other studies also revealed expanded CD90^+^ FLSs with proinflammatory features in the sublining area in RA patients [26–28]. Thus, expression of CD90 can be used to designate proinflammatory FLS subsets. Furthermore, Fan Zhang et al. [28] classified CD90^+^ FLSs into three subsets, namely, CD90^+^CD34^+^ FLSs, CD90^+^HLA-DRA^high^ FLSs, and CD90^+^DKK3^+^ FLSs. Among these subsets, CD90^+^HLA-DRA^high^ FLSs were substantially expanded and correlated with cytokine and chemokine expression in RA. The pathogenic FLS subsets in RA and their characteristic markers are summarized in Fig. 1.

## Resolving FLS subsets in RA

FLSs play various physiological functions in the homeostatic state but transform from a friend to a foe in patients with RA [42], indicating that different FLS subsets are involved in active RA and remission/homeostatic states. In 2020, Stefano Alivernini et al. [31]. analyzed FLSs from patients with active RA and those in remission. FLSs expressing MMPs can be classified into a lining-layer FLS cluster and those expressing collagens and immune mediators can be classified into four sublining-layer clusters. Although the relative proportions of these clusters were similar in patients with active RA and those in remission, their transcriptomes differed. FLS clusters in RA patients in remission expressed more mediators related to tissue repair and the resolution of inflammation. Among these subsets, CD90^+^CXCL14^+^ cells in RA patients in remission expressed high levels of GAS6, which may contribute to the regulatory functions of lining-layer MerTK^pos^ macrophages to promote the resolution of inflammation. To explore the profiles of normal FLSs and molecular networks controlling the transition from homeostatic to arthritic FLSs, in 2022, Marietta Armaka et al. [14]. performed a combined analysis of single-cell transcriptomes and epigenomes of FLSs derived from naïve and hTNFtg mice (mice that overexpress human TNF, a murine model for RA). FLSs play roles in chondrogenesis and osteogenesis, tissue repair, and immune surveillance in healthy synovium. The presence of arthritis was accompanied by reduction of homeostatic FLSs and the emergence of pathogenic FLS profiles marked by Dkk3 and Lrrc15 expression [14, 33]; these FLSs promote the inflammatory response and matrix catabolic processes. Moreover, a recent study demonstrated for the first time that FLSs have the ability to transform from proinflammatory to pro-resolving phenotypes (CD200^+^), which can reduce inflammation via interactions with ILC2 in patients with inflammatory arthritis [33]. These studies indicated the presence of resolving FLS subsets in the remission/homeostatic state that function in inflammation resolution instead of promotion; these subsets might be a new therapeutic tool to promote the resolution of inflammation and restore tissue homeostasis in patients with RA.

## Strategies for targeting pathogenic FLS subsets in RA

### Targeting cell surface markers on pathogenic FLSs

Direct depletion of pathogenic FLS subsets by targeting cell surface markers with antibodies, chimeric antigen receptor (CAR)-T cells or vaccines might be the most effective way to target pathogenic FLSs in RA. Among these FLS cell surface markers, CDH-11 first attracted attention as a promising target for RA treatment. However, a phase II trial of monoclonal antibodies targeting CDH-11 (RG6125) in RA patients was discontinued in 2018 due to a lack of efficacy [43]. Other markers, especially FAPα, are promising potential therapeutic targets for RA.

FAPα is a type II cell surface serine protease with dipeptidylpeptidase and endopeptidase activity [44] that is overexpressed in activated fibroblasts, such as those involved in cancer and fibrosis; the expression of FAPα in healthy tissues is scarce, making FAPα an attractive therapeutic target in disease [45]. Although a study in mice demonstrated that depletion of FAPα^+^ cells can result in cachexia and anemia and FAPα^+^ cells were found to reside in most tissues, including muscle and bone marrow [46], studies investigating the potential use of FAPα as a therapeutic target in diseases are ongoing. Depletion of FAPα-expressing cells by antibodies, FAP CAR-T cells and various FAP vaccines has been widely investigated in the treatment of cancer [45] and fibrosis [47, 48] and has shown safety and tolerability in phase I trials [49, 50]. Moreover, small-molecule inhibitors of FAPα (FAPIs) with high affinity and selectivity for FAPα provide new strategies to image FAPα-expressing tissues [51], as well as new treatment strategies based on linking traditional drugs with FAPα-targeted molecules [52–54]. In RA, FAPI labeled with gallium 68 (^68^Ga-FAPI) or aluminum-(18-F)-labeled 1,4,7-triazacyclononane-N, N’,N″-triacetic acid (^18^F-AIF-NOTA-FAPI) can be used to clearly reveal inflammatory joints and assess disease activity [55, 56], indicating that FAPα is an excellent candidate for RA therapy. Daphne N. Dorst [57] developed a treatment strategy for the selective destruction of FAPα^+^ cells by coupling an anti-FAP antibody with the photosensitizer IRDye700DX. This compound can accumulate in inflamed joints and induce local FAPα^+^ cell death, which moderately delayed the development of arthritis in CIA mice. A vaccine with the consensus FAPα mRNA encapsulated in a lipid nanoparticle (cFAP mRNA-LNP) prevented disease onset and arthritis development in a mouse model of RA [58]. Zinc ferrite nanoparticles (ZF-NPs) engineered to target FAPα^+^ FLSs significantly suppressed synovitis and protected against bone damage in a mouse model of RA [59]. These studies in mice further suggest that FAPα is a promising therapeutic target in RA. FAPα^+^ cell targeting studies in cancer may help us exploring new strategies for targeting FAPα^+^ FLSs in RA.

**Fig. 2:**
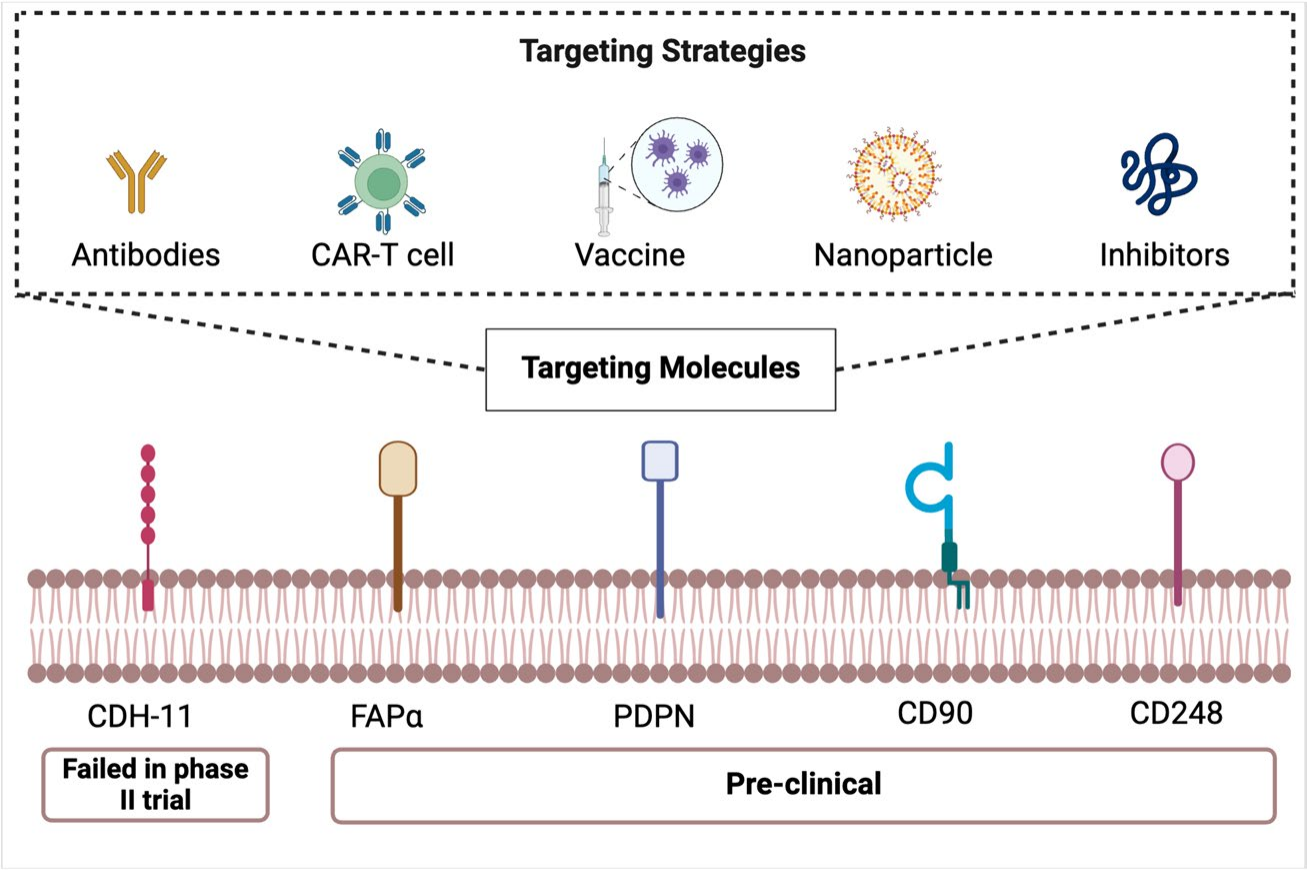
CDH-11, FAPα, PDPN, CD90 and CD248 are relatively specific synovial FLS markers. Targeting pathogenic FLS surface markers with specific antibodies, inhibitors, vaccines or CART cell might be potential strategies for the treatment of RA

The potential use of other pathogenic FLS surface markers as therapeutic targets in RA has also been investigated in animal studies. PDPN is a mucin type-1 glycoprotein with a molecular weight of 40–43 kDa. Besides on FLS, PDPN is expressed in many tumors and normal cells, especially lymphatic epithelial cells and follicular DCs [60]. PDPN has been studied in cancer as a therapeutic target by using antibodies or antagonistic peptides [61, 62]. In RA, Christopher D Buckley et al. reported in 2018 that anti-PDPN antibodies efficiently protected mice with CIA from arthritis [63]. THY-1 (CD90) is a highly N-glycosylated, glycosylphosphatidylinositol (GPI)-anchored cell surface protein, first identified for the recognition of thymoma cells, and was found to be expressed on various types of cells, such as mesenchymal stem cells (MSCs) [64]. CD90 is associated with the proinflammatory phenotype of FLSs. An anti-CD90 antibody alleviated disease progression in CIA mice by inhibiting FLS proliferation, proinflammatory cytokine release, osteoclast differentiation and angiogenesis [65], suggesting that CD90 is a potential therapeutic target for RA. CD248 is a transmembrane glycoprotein that is expressed on FLSs in the sublining area in RA. A study in CD248-deficient mice demonstrated that CD248 contributes to leukocyte accumulation and synovial hyperplasia in inflammatory arthritis, indicating that CD248 is a potential therapeutic target in RA [66]. However, the function of CD248 as a therapeutic target in RA needs to be further investigated. Potential strategies for targeting pathogenic FLS cell surface markers in RA are summarized in Fig. 2.

### Targeting signaling pathways that drive pathogenic FLS subsets differentiation

FLSs exhibit high phenotypic plasticity, and cytokines are important factors that can drive the differentiation of FLSs toward specific pathogenic RA-FLS subsets [25]; thus, cytokines can be used to modulate the FLS phenotype. TNF-α and IL-1β have been shown to stimulate fibroblasts to produce proteolytic enzymes that destroy bone and cartilage [67], and TGF-β1 can inhibit the synthesis of metalloproteinases and thereby reduce joint damage [68]. TNF-α or IL-1β can stimulate RA-FLSs to upregulate PDPN expression [25], suggesting that blockade of TNF-α or IL-1β in RA patients can inhibit the differentiation of PDPN^+^ FLSs. By using paired single-cell RNA and ATAC sequencing, multiplexed imaging, and spatial transcriptomics, along with in vitro modeling of cell-extrinsic factor signaling, a recent study revealed that myeloid and T-cell-derived TNF-α, IFN-γ, and IL-1β were important drivers of pathogenic FLS subset heterogeneity in RA [69]. Shuyang Zhao et al. [70]. showed that NK-derived IFN-γ could induce the differentiation of the inflammatory HLA-DR^+^CD90^+^ FLS phenotype, which can induce CD69 expression on CD4^+^ T cells. JAK inhibition by upadacitinib can prevent HLA-DR induction. These data indicated that JAK1 inhibition could reduce the generation of HLA-DR^+^CD90^+^ FLSs. These results may provide new insights into the mechanisms underlying the effects of cytokines and JAK inhibitors in RA.

**Fig. 3:**
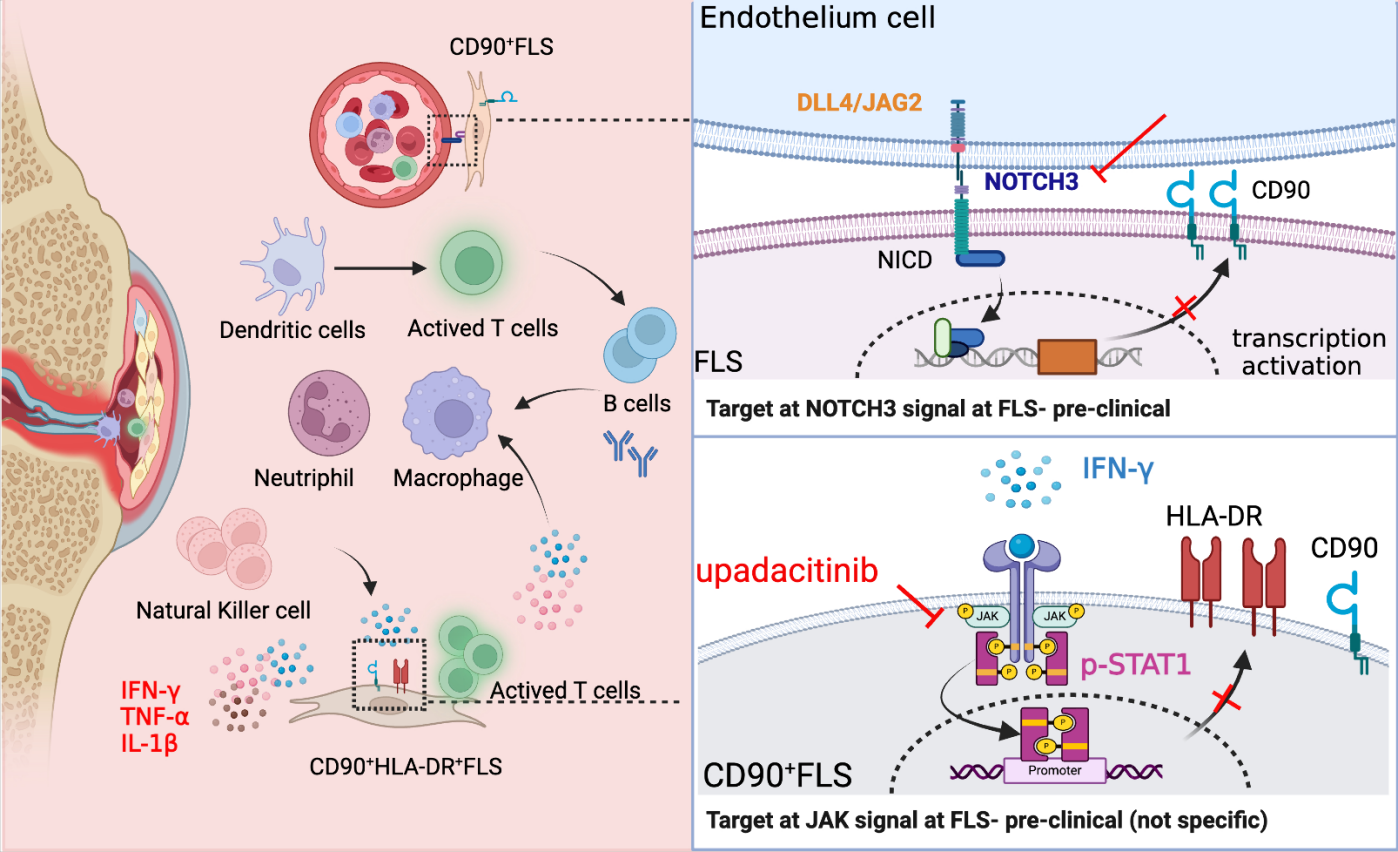
Targeting signaling pathways that drive pathogenic FLS differentiation might be a potential strategy for the treatment of RA. FLSs exhibit high phenotypic plasticity, and different cytokines or cells can stimulate the differentiation of different FLS subsets. Targeting NOTCH3 signaling at FLSs might affect CD90 expression in FLSs. IFN-γ-mediated stimulation of JAK-STAT1 signaling might also block HLA-DR expression in CD90^+^FLSs, which has proinflammatory effects during RA pathogenesis

In 2020, Kevin Wei et al. [30]. investigated the upstream signaling pathway that drives the expansion of the proinflammatory CD90^+^ FLS subset. They identified the central role of endothelium-derived Notch ligands (DLL4/JAG2) in driving the expansion of CD90^+^ sublining FLS through inductive Notch3 signaling in RA. The Notch activation signature was more enriched in CD90^high^ FLSs than in CD90^low^ FLSs, and the Notch inhibitor DAPT blocked CD90^high^ FLS differentiation. Genetic deletion or blockade of Notch3 signaling in mice relieved inflammation and protected joints in an arthritis model. LY411575 (which inhibits NOTCH-1 and the NOTCH-3 intracellular domain) suppressed inflammation and bone damage in CIA [71]. These results indicated that Notch3 is a potential therapeutic target for inhibiting the differentiation of proinflammatory FLSs in RA. Potential strategies for targeting pathogenic FLS subsets differentiation are summarized in Fig. 3.

### Restoring FLS homeostasis by promoting subsets with resolving phenotypes

Fibroblasts are complex, functional, heterogeneous cells with a wide range of effects ranging from immunosuppressive to proinflammatory effects as well as tissue repair and tissue damage effects. Thus, a delicate balance between these contradictory functions of fibroblasts may be essential for sustaining tissue homeostasis. Instead of the pathogenic phenotype of FLSs in active RA, resolving FLS clusters in RA patients in remission and immunosuppressive clusters in a homeostatic state have been demonstrated. Thus, restoring FLS homeostasis by promoting the immunosuppressive/pro-resolving and tissue repair phenotypes or switching expanded pathogenic subsets into protective subsets may be an ideal therapeutic strategy for the treatment of RA.

**Fig. 4:**
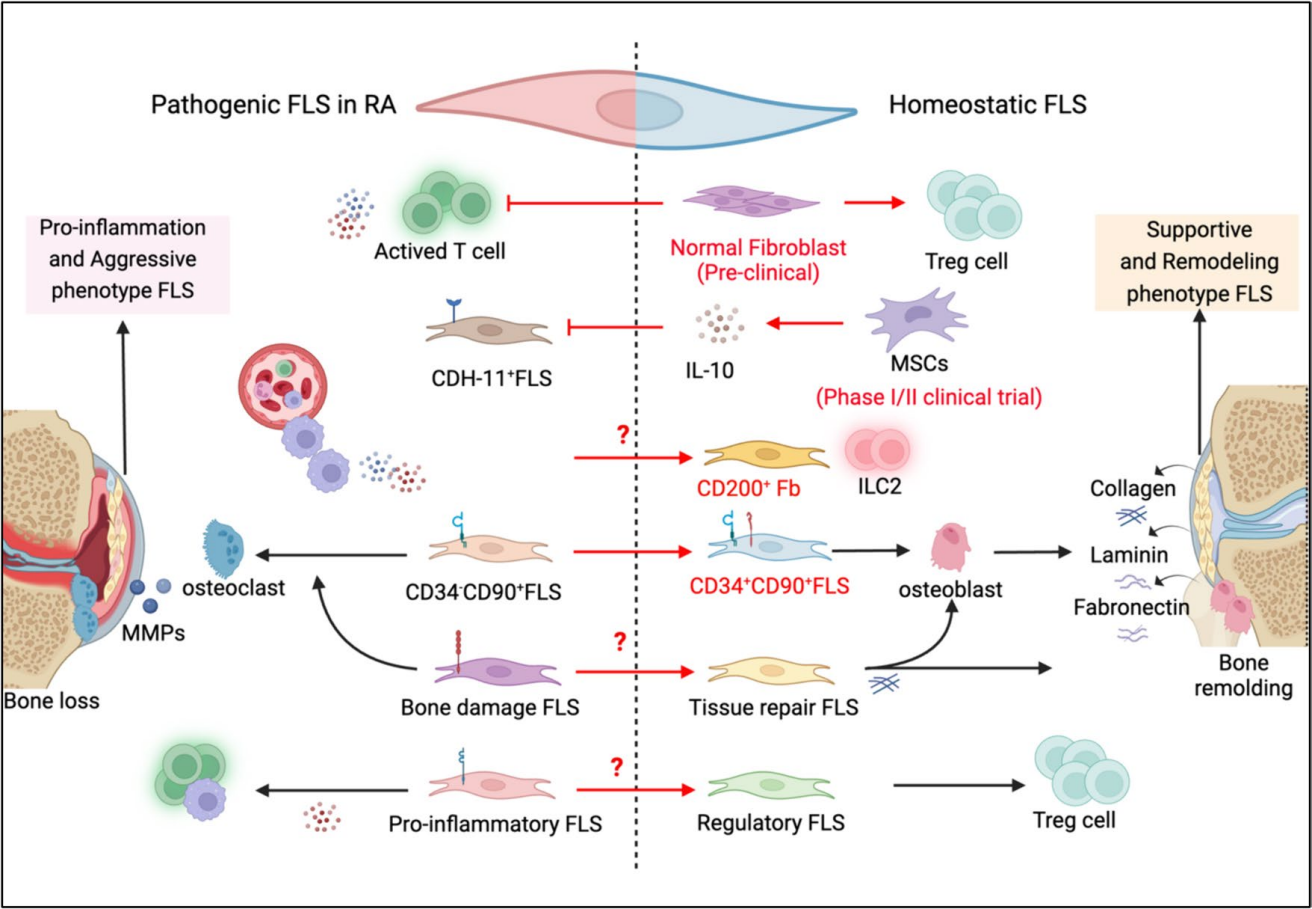
Restoring FLS homeostasis might be a potential strategy for the treatment of RA. FLS subsets in active RA patients are different from those in healthy individuals or patients in remission. Transforming proinflammatory and aggressive FLSs in RA patients into resolving or tissue repair FLSs via normal fibroblasts or mesenchymal stem cells might restore FLS homeostasis and alleviate RA

Consistent with the role of resolving CD200^+^ FLSs in mediating RA remission, a previous study also demonstrated that CD200-Fc, which can target proinflammatory cytokine expression in the joint without any obvious systemic immunosuppressive effects, is an effective therapeutic agent for CIA [72]. Umbilical cord-derived MSCs were reported to decrease CDH-11 expression in RA-FLSs, mainly by producing the anti-inflammatory cytokine IL-10 [73]. In several clinical trials of RA, such as a phase 1/2 trial (NCT03618784), promising results have been demonstrated following the treatment of joint inflammation with MSCs. Although the expanded CD34^-^CD90^+^ FLS subset in RA is considered to constitute a pathogenic phenotype, one study showed that the CD34^+^CD90^+^ FLS subset has high osteoblastic and chondrogenic potential in vitro [74]. Research on the functions of the CD34^+^CD90^+^ subgroup may lead to new treatment strategies for regenerating damaged bone/cartilage in arthritic joints. Skin fibroblasts in a homeostatic state can inhibit the proliferation of T lymphocytes. An intravenous injection of normal skin fibroblasts efficiently suppressed the severity of CIA-related inflammatory arthritis and delayed disease onset [75]. Similarly, our study showed that FLSs stimulated by IFN-γ can upregulate the expression of several inhibitory molecules, such as PD-L1 and galectin-9, on the cell membrane, which might be negative feedback mechanisms of inflammatory cytokines. FLS cell membranes with high expression of inhibitory molecules ameliorated inflammation and bone damage in CIA [76]. These data suggest that the introduction of fibroblasts with immunosuppressive/pro-resolving effects may help restore the balance of FLS subsets. However, although the driving factors of proinflammatory or tissue damaging FLS clusters in RA are beginning to be understood, the factors that induce resolving/immunosuppressive FLS subset differentiation have not been identified. Potential strategies for restoring FLS subset homeostasis are summarized in Fig. 4.

## Potential FLS-targeting strategies implemented in studies of cancer

In addition to the pathogenic effects of FLSs in RA, fibroblasts are well known for their functions in immune suppression [77] and tissue repair and play important roles in the induction of immune tolerance, inflammation resolution [78] and wound healing [79], indicating that distinct fibroblast subsets can be found in different microenvironments. Studying on fibroblast heterogeneity in other microenvironments may help us understand the mechanisms underlying FLS subset dysregulation in RA and explore new treatment strategies.

In contrast to those in RA, fibroblasts in cancer (cancer-associated fibroblasts, CAFs) exert potent immune suppression effects. CD36^+^ CAFs were recently identified as a new CAF subset with immunosuppressive effects. CD36 mediates oxidized LDL uptake to promote MIF expression, which promotes immunosuppressive MDSC accumulation and accelerates cancer progression, and CD36 inhibitors enhance the treatment efficacy of immunotherapies [80]. These findings suggest that strategies for enhancing the effects of CD36 on FLSs might be a potential therapeutic approach to reduce inflammation in individuals with RA. Among the CAF subsets in cancer [81], antigen-presenting fibroblasts, which are characterized by high expression of MHCII molecules and CD74 and can present antigens to T cells [82], were also found to be expanded in RA (CD90^+^HLA-DRA^high^ FLS) [70]; these results suggest potential new therapeutic strategies based on targeting CD74 on fibroblasts [83]. However, whether this antigen-presenting fibroblast subset can fully activate T cells or induce T-cell tolerance is still debated [84, 85]. An in-depth study on the function of antigen-presenting fibroblasts may help us better understand peripheral tolerance in RA and design methods to restore autoimmunity. Whether fibroblasts with similar phenotypes across different disease microenvironments share similar functional characteristics remains to be determined.

## Conclusions and future perspectives

FLSs play a central role in RA pathogenesis by acting as both drivers and effectors. There has been increasing interest in FLSs as important therapeutic targets in RA. FLSs are quite heterogeneous and widely arranged in different microenvironments. The increase in our understanding of pathogenic FLS clusters specific to RA has provided us with promising novel therapeutic targets. The targeting of pathogenic FLSs subsets by specific cell surface markers or upstream driving pathways may succeed in treating “non-responders” to immunosuppressive therapies, and open new RA targeting treatment era with less adverse effects associated with traditional systemic immunosuppressive therapy including csDMARDs or b/tsDMARDs.

Although high-throughput technologies have revealed several RA-specific pathogenic FLS subsets, along with the newly identified resolving FLSs in RA patients in remission, the mechanisms driving the differentiation of pathogenic or resolving subsets have not been fully elucidated. An ideal strategy for terminating persistent inflammation in individuals with RA may be restoring the homeostasis of FLS subsets by switching the pathogenic FLS phenotype to a pro-resolving/immunosuppressive phenotype. Studies on the detailed mechanisms that drive pathogenic FLS subset differentiation, including local triggers and imprinting changes, as well as the identification of factors that drive resolving/immunosuppressive fibroblast differentiation in other microenvironments, such as cancer, may help us develop treatment strategies that can restore FLS subset homeostasis. Moreover, novel therapeutic strategies that target and deliver drugs to FLSs can facilitate the development of FLS-based treatments.

## Data Availability

No datasets were generated or analysed during the current study.

## Abbreviations

RA: Rheumatoid arthritis
FLS: Fibroblast-like synoviocytes
DMARDs: Disease-modifying anti-rheumatic drugs
csDMARDs: Conventional synthetic disease-modifying anti-rheumatic drugs
b/ts DMARDs: Biological/targeted disease-modifying anti-rheumatic drugs
ECM: Extracellular matrix
MMP: Matrix metalloproteinases
PDGFRα: Platelet-derived growth factor receptor α
PDPN: Podoplanin
αSMA: α-smooth muscle actin
FAPα: Fibroblast activation protein α
UDPGD: Uridine diphosphoglucose dehydrogenase
PRG4: Proteoglycan 4
CIA: Collagen induced arthritis
CAF: Cancer associated fibroblast
